# Supplementary material for: Symptoms after COVID-19 Infection in Individuals with Multiple Sclerosis in Poland
Source: J Clin Med. 2021 Nov 10;10(22):5225. doi: 10.3390/jcm10225225 (PMC8618380; doi:10.3390/jcm10225225)
Supplement: Supplementary file 1 [file jcm-10-05225-s001.zip › jcm-1402403-supplementary.pdf]

Supplementary Materials:

Acute and residual symptoms among patients with multiple sclerosis during and after COVID-19 infection. The questionnaire.

Date:

MS Center:

Neurologist performing the examination:

Patients age:

Patients sex:

Height:

Weight:

Smoker: Yes/No

Comorbidities:

Disease course: RRMS, PPMS, SPMS

Current EDSS:

Disease duration (years):

DMT (prior to COVID-19 infection):

Duration of DMTs use (years):

Was DMT continued during SARS-Cov-2 infection:

Date of last DMT use:

1. Acute COVID-19 infection:

Date of diagnosis of the infection:

PCR result: 1-positive, 2-negative, 3-no information/ not done

Immunological test: 1-positive, 2-negative, 3-no information/ not done

Presence of antibodies: 1-positive, 2-negative, 3-no information/ not done

Contact with an infected person: 1-yes, 2-do not recall, 3-no information

First symptoms:

1-fever, 2-dry caugh, 3-fatigue, 4-sore throat, 5- headache, 6-musle pain, 7- bone and joint pain, 8-swelling of the nasal mucosa, 9- chills, 10-rasch, 11-loss of smell, 12-loss of taste, 13-dyspnoea, 14-pneumonia, 15- diarrhoea, 16-vomiting, 17-abdominal pain, 18-asymptomatic

Other:

Treatment:

WBC <3x103 : 1-yes 2-no 3-no information

Lymphocytes <1x103: 1-yes 2-no 3-no information

CRP >10 : 1-yes 2-no 3-no information

Was a chest CT scan was performed: 1-yes, 2-no, 3-no information

If the chest CT scan was performed what was the result:

Partial pressure of oxygen:1-<60%, 2->60%, 3-<95%, 4->95%

Was the patient hospitalized due to COVID-19?: 1-yes (for less than 10 days), 2-yes (over 10 days), 3-no

Did the patient required oxygen therapy during hospitalization?: 1-passiv oxygen therapy, 2-active oxygen therapy, 3-no

Did the patient recovered: 1-yes, 2-not yet, 3-the patient died due to COVID-19 infection, 4-the patient died from other causes

Additional information (cause of death):

Did the patient had symptoms 2 weeks after recovery: 1-yes, 2-no , 3-N/A

Additional relevant clinical information:

2. Residual symptoms:

Presence of residual symptoms (listed below) >4 weeks after the initial infection (but <12 weeks): 1-yes, -2-no

Presence of residual symptoms (listed below) >12 weeks after the initial infection : 1-yes, -2-no

Symptoms:

- General: 1-none, 2-fever, 3-pain, 4-fatigue

Additional information regarding symptoms above:

- Respiratory symptoms: 1-None 2- Cough 3- Dyspnoea

Additional information regarding symptoms above:

- Cardiovascular symptoms 1- None 2-Chest pain 3-Palpitations

Additional information regarding symptoms above:

- Neurological and psychiatric symptoms: 1-None, 2-Cognitive impairment ("brain fog"), 3-Disturbance of concentration of attention and memory, 4-Headaches, 5-Sleep disorders, 6-Symptoms of peripheral neuropathy [tingling and numbness], 7-Dizziness, 8-Delirium, 9-Disturbance of smell and taste, 10-Depression, 11-Anxiety, 12-other

Additional information regarding symptoms above; please name if other occurred:

- Gastrointestinal symptoms: 1-None, 2-Abdominal pain, 3-Nausea, 4-Diarrhea, 5-Appetite disorders, including anorexia

Additional information regarding symptoms above:

- Musculoskeletal symptoms: 1- None 2- Pain in muscles 3-Joint pain

3. Additional information:

Relapse: 1-up to 3 months before acute infection, 2-up to 3 month after acute infection, 3-there was no relapse

If 1 or 2 please note the date:

If yes please note the symptoms:

Note the MRI result if performed:

Methylprednisolone (i.v.) use during relapse: 1-yes , 2-no , 3- there was no relapse

If YES please note the total dose:

Oral corticosteroids use as continuation after i.v. therapy : 1-yes , 2-no , 3- N/A

If YES please note name of the drug, total dose and the date of using:

Oral corticosteroids use as treatment of relapse without i.v. therapy : 1-yes , 2-no , 3- N/A

If YES please note name of the drug, total dose and the date of using:
